# Are social security policies for Chinese landless farmers really effective on health in the process of Chinese rapid urbanization? a study on the effect of social security policies for Chinese landless farmers on their health-related quality of life

**DOI:** 10.1186/1475-9276-13-5

**Published:** 2014-01-15

**Authors:** Ying Liang, Wanyi Lu, Wei Wu

**Affiliations:** 1 Department of Social Work and Social Policy, School of Social and Behavioral Sciences, Nanjing University, 210023 Nanjing, People’s Republic of China; 2 School of Earth Science and Engineering, Nanjing University, 210023 Nanjing, People’s Republic’s of China; 3 School of Social and Behavioral Sciences, Nanjing University, 210023 Nanjing, People’s Republic of China

**Keywords:** Chinese landless farmers, Social security policy, HRQOL EQ-5D, SEM, Multinomial regression

## Abstract

**Background:**

The continuing urbanization in China has resulted in a loss of land and rights among farmers. The social security of landless farmers has attracted considerable research attention. However, only few studies measure the health-related quality of life (HRQOL) of landless farmers by employing scientific standardized scales. By using five-dimensional European quality of life (EQ-5D) scales, this study measures the HRQOL of landless farmers from a new perspective and examines how the social security policies affect their HRQOL.

**Methods:**

This study is based on a 2013 household survey that has been conducted among 1,500 landless famers who are residing in six resettlement areas in three cities within the Yangtze River Delta region, namely, Nanjing, Hangzhou, and Yangzhou. This study adopts EQ-5D scales to measure the HRQOL of these farmers. More than 50% of the respondents are in poor or non-serious health conditions, and over 50% are not satisfied with their current social security policies. The health conditions and social security policies are analyzed by multinomial regression analysis and the relationship between these two factors are analyzed via structural equation modeling (SEM).

**Results:**

*First*, the descriptive statistical analysis shows that more than 50% of the respondents are in poor or non-serious health conditions, and that the largest proportion of these farmers are suffering from anxiety or depression, which is the most serious of the five dimensions. *Second*, multinomial regression analysis shows that the satisfaction of landless farmers with their social security policies improves their living conditions, particularly in their capacity for self-care, in their ability to perform daily activities, and in the reduction of pain, anxiety, and depression. *Third*, SEM model analysis shows that the satisfaction of landless farmers with their social security policies positively influences their HRQOL. Among the five dimensions of EQ-5D, daily activities produce the greatest influence on the HRQOL of landless farmers. As regards social security policies, the land acquisition compensation policy and the employment security policy produce the greatest and weakest influences on the HRQOL of landless farmers, respectively.

**Conclusions:**

The rapid urbanization in China has deprived many farmers of their lands and of the benefits of urbanization. These farmers are often in a disadvantaged position in the land acquisition process. Statistic analysis in this paper shows that the satisfaction of landless farmers with their social security policies positively influences their HRQOL. The implementation and improvement of social security policies is very important for the long-term and sustainable development of these landless farmers.

## Introduction

Chinese economic reform has accelerated the urbanization of the country at an unprecedented pace. The Chinese urban population ratio exceeded 50% for the first time in 2011 [1]. During the 18th Congress of the Communist Party of China in 2012, terms such as “urbanization” and “urban and rural development” have been mentioned numerous times. The development process of urbanization in China has been closely observed. In the third Conference of the 18th Central Committee of the Communist Party of China in 2013, policy guidelines were proposed to improve the integration systems and mechanisms of urban and rural development and encourage farmers to participate in the modernization process and share the achievements of modern equality. The means of solving the difficulties encountered in urban development has become a primary social concern. Urbanization is the inevitable outcome of the economic development process, and a large proportion of the agricultural population has transferred to the non-agricultural sector and achieved urbanization. Modernizing in those developed countries is the universal law. More than 30 years after the policy of Reform and Opening-Up, the system arrangement of urbanization in China lifted the restriction on farmers and allowed their entry into the city. The system likewise encouraged and guided farmers to move to the city. This process has an important role in creating job opportunities, improving the industrial structure, and promoting economic growth. However, this process of rapid urbanization affects local Chinese farmers because they are forced to sell their lands to reduce the cost of urbanization. More than four million hectares of agricultural land has been bought off farmers for non-agricultural use over the past 20 years, with more than 50 million farmers losing their primary source of income. Moreover, the unreasonable land property rights system in China has significantly reduced the land use rights of these farmers [2].

These land acquisitions have shed light on the problems of landless farmers. The majority of studies focus on the four main aspects of such problems, namely, compensation and resettlement, social security, citizenship, and gender differences. *First*, although the landless farmers have been resettled by job resettlements and currency resettlements, they are unjustly compensated for their land. Areas with few or weak dynamic industries and lineage/kinship organizations are dominated by property rights that are often harmful to the livelihoods of landless farmers. The less-developed areas in inland China, especially in the Western region, are unfamiliar to non-Chinese specialists and have been given limited research attention. The imperfect compensation systems in these areas are inadequate for the dispossession of agricultural lands. Moreover, these farmers become vulnerable to poverty; they bear a significant portion of the transaction costs for urban expansion [3].

*Second*, the interests of landless farmers are not protected by an effective social security system. By the end of 2008, 1,201 counties in 27 Chinese provinces have implemented their respective social security policies for landless farmers. Although 13,240,000 landless farmers in the entire country are covered by the basic living or old-age security system [4], the beneficiaries only comprise approximately 1/4 of these farmers. Moreover, these farmers are given low compensation as they lose their lands for urban expansion. Therefore, the living conditions of approximately 60% of landless farmers have declined at varying degrees. Social tension and justice [5] as well as social insurance issues [6] have received considerable attention because of the long-term risks that they pose to the social stability and sustainable development of landless farmers.

*Third*, certain factors, such as estrangement from and fear of the city, have caused much confusion as regards the citizenship of landless farmers. Therefore, these farmers have been labeled as “landless peasants” in the retrogression of the urbanization process, and the social exclusion of these farmers is developing into a serious problem [7].

*Fourth*, although a 2008 study has observed no gender differences among the landless farmers, the population pressure and limited land resources continue to undermine the land ownership rights of women farmers [8].

By acquiring land, farmers are provided with (1) basic living security (i.e., farmers rely on their land for their basic living necessities); (2) job security (i.e., farmers rely on their land for employment) and agricultural production; (3) old-age security (i.e., farmers can sublet their lands to their children or to other individuals to support themselves during retirement); and (4) value-added security (i.e., farmers rely on their farming income to send their children to school, buy health insurance, or support their families). Losing their land will deprive these farmers of these benefits or necessities.

Therefore, certain social security policies have been implemented to support farmers after the requisitioning of their lands. The implementation of these policies allows the government to assist landless farmers with their social and economic needs, to protect their interests, to regulate their relationship, and to achieve social stability and fairness. Each province in China implements a unique social security policy, such as the Beijing—Chengdu model (comprising an urban social security system), the Qingdao model (comprising a rural social endowment insurance system), the Tianjin—Xi’an model(comprising a social insurance system), the Zhejiang—Jiangsu model (comprising a basic livelihood guarantee system), the Shanghai model (comprising a small town social security system), and the Chongqing model (comprising a commercial insurance system) [9]. All of these models provide land compensation, medical insurance, pension insurance, job security, basic living security, and housing compensation for landless farmers.

Although certain studies have examined the health conditions of landless farmers as well as their influencing factors, these studies failed to adopt a professional health measurement scale. The concept of health-related quality of life (HRQOL) and its determinants have evolved since the 1980s to encompass those aspects of overall quality of life that can be clearly shown to affect health, either physical or mental. HRQOL is related to both self-reported chronic diseases (diabetes, breast cancer, arthritis, and hypertension) and their risk factors (body mass index, physical inactivity, and smoking status). Measuring HRQOL can help determine the burden of preventable diseases, injuries, and disabilities. HRQOL can likewise provide valuable new insights into the relationships between HRQOL and risk factors, and assist in monitoring the progress to achieve the health objectives of a nation [10]. The interpretation and publication of these data can garner support for health policies and legislation, help allocate resources based on unmet needs, guide the development of strategic plans, and monitor the effectiveness of broad community interventions [11].

Studies in HRQOL and related factors of populations in mainland China have flourished in recent years. Chan et al. conducted a cross-sectional study on the HRQOL of depressed Chinese older people in Shanghai [12]. Liu and Guo measured loneliness and HRQOL for the empty nest elderly in the rural area of a mountainous county in China [13]. Wang et al. measured HRQOL of populations in Shanghai using the 36-item Short Form (SF-36) [14]. Lam et al. studied HRQOL of Southern Chinese with chronic hepatitis B infection [15]. Wang et al. attempted to find the effect of hypertension on HRQOL of population in Shanghai [16]. Liang and Wang developed a new perspective to study the HRQOL of survivors of Sichuan earthquakes in China, and studied the effect of post-earthquake policies on the HRQOL of survivors [17].

The five-dimensional European quality of health scale (EQ-5D) is a handy, easy-to-use tool for measuring health outcomes that has been increasingly used in health services, economic analyses, pharmacies, and population health surveys. This scale can be used to calculate for quality-adjusted life-years [18], which in turn can be used to assess and test the quality and effectiveness of health services. Taiwanese version of the EQ-5D appears to be moderately valid and reliable too for measuring the health status of the general population in Taiwan [19]. Besides, data provide promising evidence for the measurement equivalence of English and Chinese EQ-5D versions in Singapore [20]. This tool has also been adopted by several studies on mainland China [21-23], such as the National Health Services Survey in 2008. By adopting the EQ-5D scales, Sun S. et al. examined the health status of people in the mainland by age, sex, and socio-economic status [24] as well as the regional differences in terms of health status [25], Sun X. et al. studied the relationship between the living conditions and the HRQOL of urban seniors [26], and He et al. studied the HRQOL of nurses and doctors [27].

Numerous studies have also examined the influencing factors on health of Chinese farmers. Wang HM et al. investigated the influences of trust and distrust (cognitive dimension of social capital) on health in rural China [28], Wang H. et al. examined the influence of rural mutual health service on health [29], and Mou et al. examined the health conditions and depressive symptoms of migrant factory workers in Shenzhen [30]. Many studies have also adoptedEQ-5D to measure the HRQOL of different groups that suffer from certain affliction, such as rheumatic diseases [31], chronic prostatitis/chronic pelvic pain syndrome [32], Kashin–Beck disease [33]. Another study also investigated individuals who are and are not suffering from chronic diseases [34]. An additional study using EQ-5D evaluation indicates that the method could be enhanced when compared against utility measurements certain diseases [35].

The majorities of studies have only focused on the health conditions of Chinese rural-to-urban migrants [36-40], and have rarely adopted the EQ-5D scale to measure such conditions. The health conditions and HRQOL of Chinese landless farmers have not been investigated by standardized scales. Therefore, this paper addresses a new research area. This paper also investigates this topic from a new perspective by adopting the EQ-5D scale to measure the HRQOL of landless farmers and by examining the influence of social security policies on their HRQOL. Landless farmers suffer the most during the urbanization of the country, particularly in terms of their quality of life, employment, and pension. This issue has become a major problem in the development of the Chinese economy and society. Many domestic studies have suggested social security policies and proper measures for the placement of landless farmers. Helping these farmers maintain their livelihoods can positively affect the long-term development, stability, and prosperity of society. Therefore, the social security policies for landless farmers in China must be improved as soon as possible.

This paper studies the effects of social security policy for Chinese landless farmers on HRQOL from a new perspective. It offers a two-fold contribution. *First*, this paper comprehensively measures the satisfaction of landless farmers on the social security policies that are being offered to them by the Chinese government. These policies cover the land compensation, medical insurance, pension, job security, basic livelihood security, and housing compensation of landless farmers. *Second*, this study adopts the EQ-5D scale for the quantitative analysis of the health conditions and HRQOL.

Farmers lose much more during the requisitioning of their lands. They lose their rights and interests; they dissociate themselves from certain identities, such as “peasants”, “citizen”, “urban”, and “rural”. However, these farmers cannot enjoy the same social security policies that urban residents are being provided, which increases their vulnerability to poverty and oppression. This paper aims to (1) assess the HRQOL of landless farmers as well as their satisfaction with present social security policies, (2) to examine if the Chinese social security policies for landless farmers can help improve the HRQOL of their intended recipients, and (3) to provide certain suggestions for the improvement of these policies.

## Methods

### Sampling

The Yangtze River Delta is an alluvial plain that lies before the mouth of the Yangtze River, which has become the largest economic zone of the country, the strongest economic center in the Asia-Pacific region, and an advanced manufacturing base for international countries. The Yangtze River Delta has been recognized as one of the six globally competitive city groups in China, and is aiming to become the largest metropolitan area in the world by 2018. It is one of the typical representative regions in the rapid urbanization process of China.

**Figure 1:**
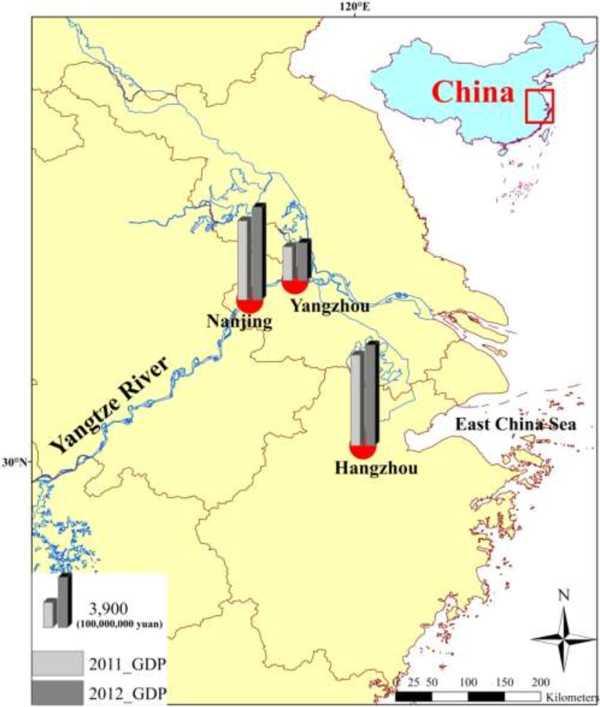
**Geographical location and GDP in 2011 and 2012 of three sample cities.** Data were obtained from the local bureau of statistics of Yangzhou, Nanjing, and Hangzhou. The URLs are http://tjj.yangzhou.gov.cn/, http://www.njtj.gov.cn/, and http://www.hzstats.gov.cn/web/ separately.

As an important part of the Yangtze River Plain, the Yangtze River Delta is located east of Zhenjiang, Jiangsu Province, north of Hangzhou Bay, and south of Tongyang canal. The river runs from the Tongyang canal to the Hangzhou Bay and from Nanjing to the seaside, which includes Shanghai, southern Jiangsu, northern Zhejiang, and the neighboring sea area. The area is approximately 99,600 km^2,^ and is predominantly a great plain. After the expansion of the State Council, the Yangtze River Delta region comprises 30 cities, 210,000 km^2^ land area, and 159 million residents. The region has a flat shoreline, yellow and turbid seawater, and an intertidal shoal that ranges from a few kilometers to tens of kilometers. The cities in this region specialize in different areas, including industry, finance, trade, education, science, and culture. The economic strength and specialties of these cities are crucial in driving the economic development of the Yangtze River region, in connecting the domestic and foreign markets, in attracting foreign investment, in promoting industry and technology transfer, in competing in the international market, and in promoting regional reorganization (Figure 1).

The rapid economic development of the Yangtze River Delta is accompanied by its rapid urbanization. Therefore, this region presents an ideal setting for a survey of landless farmers in the rapid process of Chinese urbanization. *First*, three cities, namely, Yangzhou, Nanjing, and Hangzhou, have been selected out of the 30 cities in the region via a purposive non-random sampling method. The economic development levels of these cities have recently catapulted from low to high, which is similar to the socio-economic development in eastern Central China. These cities have witnessed a 10% gross domestic product (GDP) growth in 2012 and have experienced a rapid development over the recent years [41]. Carry Sampling has been applied in these regions to select a more representative sample and to devise highly effective suggestions for the improvement of the HRQOL of landless farmers.

*Second*, according to the Ministry of Civil Affairs, the landless farmers are relocated in communities after they receive their land compensation. The simple random sampling method is adopted to select two resettlement communities for landless farmers from each of the selected cities. At the end of the sampling, the Jinyangyuan and Huapinyuan communities in Yangzhou, the Yinxing and Lingshun communities in Nanjing, and the Jialv and Tongren communities in Hangzhou have been selected for the survey.

*Third*, professionally trained investigators have conducted household surveys on landless farmers that reside within the selected communities. The households are stratified based on the number of adults. The Kish Grid sampling method is adopted for the equal probability sampling within each stratum. The recorded age and gender in the Kish Grid sample are equivalent to the reported population in the China health survey, which attests to the practicability of the Kish Grid method. The Kish Grid can also decrease the bias in the sampling of households [42].

The survey questionnaires mainly focus on the HRQOL of landless farmers as measured by EQ-5D. Each respondent completes the questionnaire under the supervision of the investigators. Out of the 1,500 distributed questionnaires (500 questionnaires to each of the three cities), 1,236 valid questionnaires have been returned, achieving an 82.4% response rate.

### Variables

#### Independent variables

EQ-5D is a universal and standardized scale that has been developed by five European countries in 1987. This tool can perform a cross-cultural comparison and measure general HRQOL by drawing comprehensive scores from each unit by multidimensional utility policy. The respondents can easily understand EQ-5D given the simplicity and intuitiveness of the scale. This scale is commonly used in health screenings, health factors analyses, and disease severity comparisons.

EQ-5D measures HRQOL based on five dimensions, namely, mobility, self-care ability, daily activities, pain/discomfort, and anxiety/depression. The first two dimensions reflect the physical health, daily activities reflect the social function, and pain/discomfort and anxiety/depression reflect the mental health of the respondents. The respondents can choose among three options for each dimension, namely, no problems, moderate problems, and serious problem. Three levels and five dimensions of health measurement policy are formed from the scale, and a five-digit figure is produced to reflect the current health status of the respondents. For example, “11,111” means that the respondents do not have any problems in the five dimensions, whereas “33,333” means that the respondents have serious problems in the five dimensions. As many as 243 combinations can be generated from this process, which can be given as many as 245 interpretations, including the two special cases of unconsciousness and death.

The description of the results indicates that the options for the five dimensions are distributed averagely with no special abnormal data occurring. Less than 42% of the respondents do not have any problems on all five dimensions. Therefore, more than half of the respondents are in poor condition or with serious health conditions, particularly in the dimensions of mobility, self-care ability, and daily activities.

No significant difference has been observed in the mean and standard deviation of each dimension. The majority of respondents are shown to experience anxiety/depression, which reflects the poor psychological state of the landless farmers. The results for each of the five dimensions are shown as follows. The results of descriptive analysis of variable are presented (see Table 1):

(1) Mobility. The number of valid cases is 1,220. A total of 41.7% of the respondents do not have any mobility problems, 31.8% cannot rise from their beds, and 26.5% cannot move easily.

(2) Self-care. The number of valid cases is 1,194. A total of 39.6% of the respondents can take care of themselves without any difficulty, 26.0% encounter difficulties in brushing their teeth, bathing, or dressing, and 34.3% cannot take care of themselves.

(3) Daily activities. The number of valid cases is 1,213. A total of 36.4% of the respondents have no problems in performing their daily activities, 32.2% encounter difficulties in performing their daily activities, and 31.4% cannot perform their daily activities.

(4) Pain/discomfort. A total of 37.7% of the respondents do not feel any pain or discomfort, 27.3% feel moderate pain or discomfort, and 35.0% feel extreme pain or discomfort.

(5) Anxiety/depression. A total of 37.5% of the respondents do not experience anxiety or depression, 26.5% occasionally experience anxiety or depression, and 36.1% experience serious anxiety or depression.

**Table 1:** Descriptive analysis of the variables

		**Grades and answers**	**%**	$\bar{x}\pm s$	**Skewness**	**Standard error of skewness**	**Kurtosis**	**Standard error of kurtosis**
Independent variables	Mobility	1 = I can walk anywhere without any problems	41.7	1.90 ± 0.852	0.191	0.070	−1.597	0.140
		2 = I have some problems in mobility	26.5					
		3 = I cannot get up	31.8					
	Self-care	1 = I can take good care of myself without any problems	39.6	1.95 ± 0.859	0.101	0.071	−1.637	0.141
		2 = I have some problems in washing my face, brushing my teeth, bathing, and dressing	26.0					
		3 = I cannot wash my face, brush my teeth, bathe, and dress	34.3					
	Daily activities	1 = I can perform my daily activities without any problems	36.4	1.95 ± 0.822	0.092	0.070	−1.515	0.140
		2 = I have some problems in performing my daily activities	32.2					
		3 = I cannot perform my daily activities	31.4					
	Pain/discomfort	1 = I do not feel any pain/discomfort	37.7	1.97 ± 0.852	0.051	0.070	−1.623	0.141
		2 = I feel moderate pain/discomfort	27.3					
		3 = I feel extreme pain/discomfort	35.0					
	Anxiety/depression	1 = I do not feel anxiety/depression	37.5	1.99 ± 0.858	0.027	0.071	−1.641	0.141
		2 = I feel moderate anxiety/depression	26.5					
		3 = I feel serious anxiety/depression	36.1					
Dependent variables	Policy1	Land acquisition compensation policy		2.82 ± 1.325	0.199	0.071	−1.118	0.142
	Policy2	Medical insurance policy		2.76 ± 1.457	0.253	0.070	−1.351	0.140
	Policy3	Pension insurance policy		2.68 ± 1.396	0.438	0.070	−1.138	0.140
	Policy4	Employment security policy		2.78 ± 1.435	0.229	0.070	−1.352	0.140
	Policy5	Basic livelihood guarantee policy		2.86 ± 1.346	0.226	0.071	−1.213	0.141
	Policy6	Housing compensation policy		2.65 ± 1.476	0.279	0.070	−1.395	0.139

Previous studies showed high responsiveness for both the EQ-5D and the SF-36, indicating that both instruments are suitable for use as outcome measures in clinical trials in elderly hip fracture patients [43]. Subjects with musculoskeletal diseases had significantly lower scores on all SF-36 dimensions than those without musculoskeletal disease. Similar results were found for EQ-5D [44]. Some models mapping the SF-36 onto the EQ-5D have similar predictions across inpatient and outpatient setting and medical conditions [45]. Health outcome assessment within routine health care seems to be acceptable, and even appreciated, by patients. Questionnaire length and ease of response were not found to be crucial arguments in choosing between SF-36 and EQ-5D [46]. In this study, brief EQ-5D scale is more convenient and suitable for special vulnerable group of landless -farmers. As the Chinese landless farmers have lower level of education, using the EQ-5D scale, which contains fewer questions and is easier to get understood, would be more appropriate. In addition, the sampling size in this study (n = 2,000) is relatively large, and three different cities are sampled. Using the EQ-5D scale would be more economical, convenient, and easier to operate.

**Figure 2:**
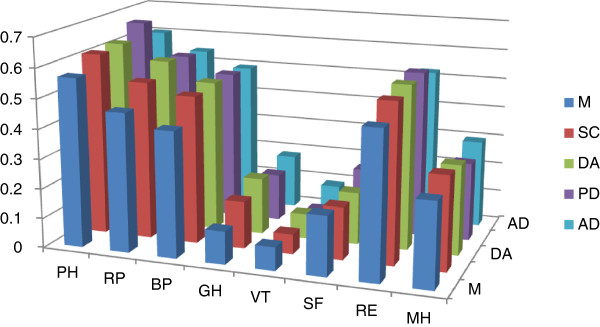
**Correlations between the EQ-5D and SF-36.** Notes: the outcome is the correlations between scores of EQ-5D and SF-36. In SF-36, PH refers to physical functioning; RP refers to role-physical; BP refers to bodily pain; GH refers to general health; VT refers to vitality; SF refers to social functioning; RE refers to role-emotional; MH refers to mental health. In EQ-5D, M refers to mobility; SC refers to self-care; DA refers to daily activity; PD refers to pain/discomfort; AD refers to anxiety/depression.

We compare EQ-5D scale with SF-36 scale (Figure 2). The result shows that, each dimensions of EQ-5D and SF-36 are strongly correlated, especially in the PH, RP, BP and RE areas; low degree of correlations is between GH, VT and EQ-5D scores. In the similar areas, comparison between scales is more appropriate. EQ-5D did not involve issues related to GH and VT, so we get lower correlation results. Among them, the correlation between PH and PD was 0.665 (P = 0.000); correlation between PH and DA was 0.620 (P = 0.000). Overall, high validity of EQ-5D can well reflect the HRQOL of landless farmers.

### Dependent variables

The social security policies for landless farmers are used as dependent variables. Respondents are asked about their perceptions toward such policies. The subjective feelings of farmers toward such policies can be helpful in devising suggestions for the improvement of existing policies and for the implementation of new, highly effective policies.

In 2006, the General Office of the State Council forwarded the notice on improving employment training and social security work for landless farmers. The notice was released by the Ministry of Labor and Social Work to the local government [47]. This official document regarded the improvements in employment training and social security work for landless farmers as vital to land requisition system reform. The document likewise asked the government to clearly define the scope and attain focus and balance, as well as to implement classification guidance both within and outside the urban area in accordance with different situations. This study focuses on six policies, as follows:

(1) Land acquisition compensation policy

Land acquisition refers to the transferring of land ownership between the state and the farmers. In accordance with the 2004 amendment to the Chinese constitution, the land acquisition must be of national interest, must be in accordance with the law, and must be approved by the authorities. The compensation for farmers can be given collectively or individually.

(2) Medical insurance policy

Landless farmers are also provided with basic medical insurance as part of their compensation to cover their medical expenses and to prevent illness-induced poverty. Landless farmers who reside outside urban areas are covered by the new rural cooperative medical care insurance, whereas those who are living with in urban areas are under this same coverage. The implementation of this insurance abides by the new cooperative medical care and basic medical insurance policy for urban residents.

(3) Pension insurance policy

The pension insurance policy gives the landless farmers a sense of security as they grow older. Elderly farmers can receive a basic pension, which can be adjusted periodically. This policy reduces the burden of farmers with regard to the education of their children and the wellbeing of their families. The economic situation of the country may significantly affect the provision of pensions, given that the burden of the nation is massive as compared to the burden of individuals. The basic endowment insurance policies for landless farmers are financed by the farmers themselves, by the farmers as a collective, and by the Chinese government. The implementation of this policy requires coordination among cities. Counties manage their fiscal revenue and expenditure accounts as well as balance their budget for pensions. Beginning from December 31, 2008, farmers who satisfy the conditions that are set by their respective counties can apply for such policy. In 2013, many provinces introduced new methods of pension insurance policies. For instance, the Shandong government merged the old rural and urban pension insurance policies into a unified system for all residents. Landless farmers are included in the resident pension insurance system. The Jiangsu government identified four principles, and the participation of landless farmers in the social security system is mandatory [48].

(4) Employment security policy

Counties and cities must provide their resident landless farmers who are within the labor age with an urban employment policy, job training and mentoring, and employment opportunities. The government must actively develop careers in public service sector for landless farmers who are having difficulty in finding employment. The government must also supervise and require certain institutions to prioritize landless farmers when expanding their workforce. Job placement may occur for farmers who meet the conditions of labor. In addition, land units and employment services can enable landless farmers to conduct contracts with tripartite commission.

(5) Basic livelihood guarantee policy

Landless famers who are living within urban areas and are earning below the minimum living standard can apply for the minimum living allowance policy. Landless farmers who are able to secure medical insurance can also apply for this policy.

(6) Housing compensation policy

The current relocation compensation for landless farmers can be classified into relocation compensation, monetary compensation, property swap, and farmers self-financing. Homesteads in exurban and rural areas are usually relocated and rearranged. However, urban and villages have no separate arrangement for the relocation of homesteads. Housing compensation primarily takes the form of monetary or in-kind compensation, in which the farmers can receive monetary payment to buy a new house or receive housing from the government, respectively.

To prevent ceiling effect, each policies are evaluated on a five-point scale, namely, 1 = very poor, 2 = poor, 3 = average, 4 = relatively good, and 5 = very good. The majority of respondents have given “very poor” and “poor” ratings to these policies. A total of 32.8% of the respondents have answered that the implementation of the housing compensation policy is very poor, whereas 17.4% have answered that the implementation of the medical insurance policy is very good.

### Analysis method

#### Validity analysis

AMOS and MPLUS software are used to analyze the variance and covariance among the variables. The results of the analysis are presented in Table 2.

**Table 2:** Covariance and Spearman correlation coefficients

	**Estimate**	**S.E.**	**C.R.**	**P**	**Spearman correlation coefficients**
Self-care ← mobility	0.335	0.022	14.917	***	0.488**
Mobility ← daily life	0.323	0.022	14.745	***	0.476**
Mobility ← pain	0.341	0.023	14.941	***	0.478**
Mobility ← depression	0.320	0.023	14.078	***	0.448**
Self-care ← daily life	0.352	0.022	15.850	***	0.524**
Self-care ← pain	0.362	0.023	15.727	***	0.517**
Self-care ← depression	0.347	0.023	15.078	***	0.488**
Daily life ← pain	0.343	0.022	15.327	***	0.498**
Daily life ← depression	0.352	0.023	15.523	***	0.508**
Pain ← depression	0.349	0.023	14.949	***	0.482**

Five variables pass the significance test (covariance coefficient ≥0.320, and C.R. coefficient >14.0), which attests to the validity of the scales and the reliability of the results. The Spearman correlation coefficient is used to analyze the validity of theEQ-5D scale. The positive correlation coefficients indicate a positive relationship between the variables. The correlation coefficients between the variables are insignificant and are all greater than 0.44, which support the validity of the scales and reflect the HRQOL of the landless farmers. For example, the correlation coefficient between self-care and mobility is 0.488, which indicates a moderate correlation between the two variables and that the use of these variables to predict each other can reduce errors by 48.8%. Self-care and daily activities have obtained the highest correlation coefficient of 0.524, which indicates that the self-care ability to predict the daily activities of landless farmers and vice versa can reduce the errors by 52.4%.

### Regression analysis

Multinomial regression analysis is adopted to analyze the relationship between the social security policies and HRQOL of landless farmers. Table 3 shows five models, whose dependent variables are divided into three categories. For example, mobility is divided into “1 = I can walk anywhere without any problems”, “2 = I have some problems in mobility”, and “3 = I cannot get up”. Multinomial regression analysis is adopted given that the categories are not arranged in a particular order. In the multinomial logistic regression model (MNLM), the coefficients are estimated based on each dependent variable. The number of parameters to be estimated is calculated as K(J–1), where K is the number of independent variables and J is the number of categories in the dependent variable. If one independent variable and four categories of the dependent variable categories occur, the number of estimated parameters is calculated as 1 (4–1) = 3. Table 3 shows six X variables and three categories of the Y variable. Therefore, the number of estimated parameters is computed as 6(3–1) = 12. The estimated parameters represent the logit coefficients that indicate the independent log odds of each X variable to be included in the interest or contrast categories of the dependent variable. For example, the last category of Model 1 (“3 = I cannot get up”) is given a coefficient of 0.

**Table 3:** Multinomial regression analyses

	**Model1: Mobility**			**Model2: Self-care**			**Model3: Daily activities**			**Model4: Pain/discomfort**			**Model5: Depression/anxiety**		
	**B**	**Wald**	**Exp(B)**	**B**	**Wald**	**Exp(B)**	**B**	**Wald**	**Exp(B)**	**B**	**Wald**	**Exp(B)**	**B**	**Wald**	**Exp(B)**
Intercept	−4.168***	223.029		−5.035***	262.344		−5.124***	256.522		−4.269***	236.402		−4.550***	245.650	
Policy1	.265**	9.275	1.304	.331***	13.540	1.393	.360***	14.845	1.194	.452***	28.114	1.571	.259**	8.857	1.296
Policy2	443***	31.147	1.557	.357***	19.384	1.429	.385***	20.377	1.243	.134	3.025	1.143	.212**	7.393	1.236
Policy3	.416***	22.808	1.516	.294***	11.531	1.341	.231*	6.274	1.051	.282***	11.831	1.326	.278**	11.204	1.321
Policy4	.256**	9.973	1.292	.220**	7.025	1.246	.275***	10.288	1.113	.290***	13.718	1.337	.208*	6.650	1.231
Policy5	.063	0.462	1.065	.440***	20.741	1.553	.438***	19.013	1.273	.254**	7.928	1.289	.321***	12.240	1.378
Policy6	.253**	9.535	1.288	.262**	9.767	1.299	.304***	12.192	1.142	.175*	4.828	1.191	.397***	24.214	1.487
Intercept	−1.895***	57.409		−2.042***	64.441		−2.390***	84.768		−1.795***	57.620		−1.741***	53.338	
Policy1	.123	2.095	1.131	.119	2.022	1.127	.185*	4.849	1.021	.210**	6.581	1.234	.157	3.726	1.170
Policy2	.080	1.008	1.084	.219**	7.940	1.245	.259***	10.822	1.110	.003	.002	1.003	.119	2.517	1.127
Policy3	.241**	7.422	1.273	.055	.418	1.057	.144	2.773	.975	.167*	4.144	1.182	.122	2.185	1.129
Policy4	.179*	5.111	1.196	.074	.890	1.077	.023	.088	.877	.051	.441	1.052	.038	.238	1.038
Policy5	-.005	0.003	0.995	.206*	5.027	1.229	.344***	14.090	1.179	.174*	3.954	1.190	.147	2.828	1.159
Policy6	.150	3.373	1.161	.122	2.368	1.130	.117	2.140	.961	.054	.477	1.055	.050	.412	1.052
−2log likelihood	2.065E3			2.070E3			2.090E3			2.078E3			2.069E3		
−2log likelihood	1.602E3			1.546E3			1.585E3			1.661E3			1.605E3		
chi-square	462.917			523.922			505.257			416.901			463.291		
Df	12			12			12			12			12		
Sig	0.000			0.000			0.000			0.000			0.000		

Note 1: ***P < 0.001, **P < 0.01, *P < 0.05.

Note 2: Policy 1: Land acquisition compensation policy.

Policy 2: Medical insurance policy.

Policy 3: Pension insurance policy.

Policy 4: Employment security policy.

Policy 5: Basic livelihood guarantee policy.

Policy 6: Housing compensation policy.

Multinomial regression models:

$$\mathrm{Log}\mathrm{it}\left(p_{1}\right)=\alpha_{1}+\beta_{11}x_{1}+\ldots +\beta_{16}x_{6}$$

and

$$\mathrm{Log}\mathrm{it}\left(p_{2}\right)=\alpha_{2}+\beta_{21}x_{1}+\ldots +\beta_{26}x_{6},$$

Where P1= $\frac{\pi_{1}}{\pi_{3}}$, and P2= $\frac{\pi_{2}}{\pi_{3}}$, π1, π2 and π3 represent the dependent first category, the dependent second category, and the probability values of the third category. The sum of these three values is 1.Themodeluses the third category of each dependent variable as the reference category, X as the social security policy, and βis as the estimated parameters.

The Exp(B) value in Table 3 refers to the relative risk ratio. When Exp(B) is greater than 1, the probability for an event to occur is higher, or the independent variable is positive to the event. Otherwise, the probability for an event to occur is lower, or the independent variable is negative to the event.

### Structural equation modeling (SEM)

The SEM model combines the observed data with the ideal model to examine how the former supports the latter. This model tests the relationship between the observed and latent variables and vice versa, with a high testing potency. This model is commonly adopted to explore the structure of the social, psychological, and relational models. Before examining the relationship between the social security policies and HRQOL of landless farmers, STATA is used to pre-treat the collected data and the missing data are replaced by the mean of the present data.

### Ethical statement

Research involving human subjects (including human material or human data) in this study has been performed with the approval of ethnics committee of the School of Social and Behavioral Sciences, Nanjing University. Research carries out on humans has been in compliance with the Helsinki Declaration. And the authors would take the interpretation and responsibility for results involving human subjects in this study.

## Results

### Regression analysis

#### Model 1

(1) Degree of fitting

The mobility of the landless farmers and the social security policies are used as the dependent and independent variables, respectively. In the model, Cox and Snell R2 =0.345, Nagelkerke = 0.391, and Sig = 0.000, which indicate an acceptable degree of fitting.

(2) Significance

Based on the p value of model 1, all policies, except for the basic livelihood guarantee policy, are significantly related to “I can walk anywhere without any problems”, whereas the pension insurance and employment security policies are significantly related to “I have some difficulties in my mobility”.

(3) Exp(B)

As regards pension insurance policy, the Exp(B) of “I can walk anywhere without any problems” is 1.156 times greater than that of “I cannot get up”, and the Exp(B) of “I have some problems in mobility” is 1.237 times greater than that of “I cannot get up”, which shows that the mobility of the respondents are improved as they become more satisfied with the pension insurance policy. As regards basic livelihood guarantee policy, the Exp(B) of “I have some problems in my mobility” is 0.995 times (<1) greater than “I cannot get up”, which shows that the satisfaction of the respondents with this policy cannot improve their mobility. This result is expected, given that mobility is an objective condition that may be related with congenital or acquired disability.

#### Model 2

(1) Degree of fitting

The self-care and social security policies are used as dependent and independent variables, respectively. In the model, Cox and Snell R2 =0.388, Nagelkerke = 0.438, and Sig = 0.000, which show an acceptable degree of fitting. The last category of self-care ability, “3 = I cannot wash my face, brush my teeth, bathe, or dress” is selected as the reference category in STATA by default.

(2) Significance

All policies are significantly related to “I can take good care of myself without any problems”. Only the pension insurance and basic livelihood guarantee policies are significantly related to “I have some problems in washing my face, brushing my teeth, bathing, or dressing”.

(3) Exp(B)

The Exp(B) of all categories is greater than 1, which shows that the dependent variables are positive to the events. The satisfaction of landless farmers with the present policies can improve their self-care ability, given that such satisfaction can induce positive feelings from the landless farmers, hence motivating them to take care of themselves amid certain difficulties.

#### Model 3

(1) Degree of fitting

The daily activities and social security policies are used as dependent and independent variables, respectively. In the model, Cox and Snell R2 =0.372, Nagelkerke = 0.419, and Sig = 0.000, which imply an acceptable degree of fitting. The last category, “3 = I cannot perform my daily activities”, is used as the reference category.

(2) Exp(B)

The satisfaction of the landless farmers with the pension insurance, employment security, and housing compensation policies cannot improve their ability to perform their daily activities. As regards the pension insurance policy, the Exp(B) of “I have some difficulties in performing my daily activities” is 0.975 times (<1) greater than that of “I cannot perform my daily activities”. As regards the employment security policy, the Exp(B) of “I have some difficulties in performing my daily activities” is 0.975 times (<1) greater than “I cannot perform my daily activities”. As regards the housing compensation policy, the Exp(B) of “I have some difficulties in performing my daily activities” is 0.961 times (<1) greater than “I cannot perform my daily activities”.

The satisfaction of landless farmers with the pension insurance policy results from the improvements in the implementation and operation of this policy. This policy can ease the financial burden and disease-induced suffering of the landless farmers, which ultimately improves the ability of the landless farmers to perform their daily activities. The employment security and housing compensation policies in directly eases the economic pressure on the landless farmers, which can further be improved by adequate healthcare. The satisfaction of the landless farmers with these policies lessens their concerns on their difficulty to perform their daily activities.

#### Model 4

(1) Degree of fitting

The pain/discomfort and the social security policies are used as dependent and independent variables, respectively. In the model, the Cox and Snell R2 =0.30, Nagelkerke = 0.361, and Sig = 0.000, which indicate an acceptable degree of fitting. The last category, “3 = I feel extreme pain/discomfort” is used as the reference category in the STATA by default.

(2) Significance

All policies are significantly related to “I do not feel any pain/discomfort”. The land acquisition compensation, pension insurance, and basic livelihood guarantee policies are significantly related to “I feel moderate pain/discomfort”.

(3) Exp(B)

The Exp(B) of all categories is greater than 1, which indicates that the satisfaction of the landless farmers on the social security policies positively affects their pain/discomfort. From a subjective angle, these security policies can ease the pain of the landless farmers, whereas from an objective point of view, these policies can prevent the occurrence of pain/discomfort among the subjects.

#### Model 5

(1) Degree of fitting

The anxiety/depression and the social security policies are the dependent and independent variables, respectively. In the model, Cox and Snell R2 =0.351, Nagelkerke = 0.396, and Sig = 0.000, which indicate an acceptable degree of fitting. The last category, “3 = I feel serious anxiety/depression”, is used as the reference category.

(2) Significance

All policies are significantly related to “I do not feel anxiety/depression”, whereas none of these policies are significantly related to “I feel moderate anxiety/depression”, which implies that the social security policies produce a positive effect on landless farmers with a healthy psychological state.

(3) Exp(B)

The Exp(B) of all categories is greater than 1, which implies that the landless farmers who are highly satisfied with the policies are in a healthy psychological state.

The multinomial regression analysis shows that the social security policies can significantly affect the self-care, daily activities, pain/discomfort, and anxiety/depression of the landless farmers. Such effects are highly prevalent among famers with positive HRQOL and less pronounced among farmers with negative HRQOL. Although social security can improve the HRQOL of farmers, the extent of such effects may vary among different groups.

## SEM results

**Figure 3:**
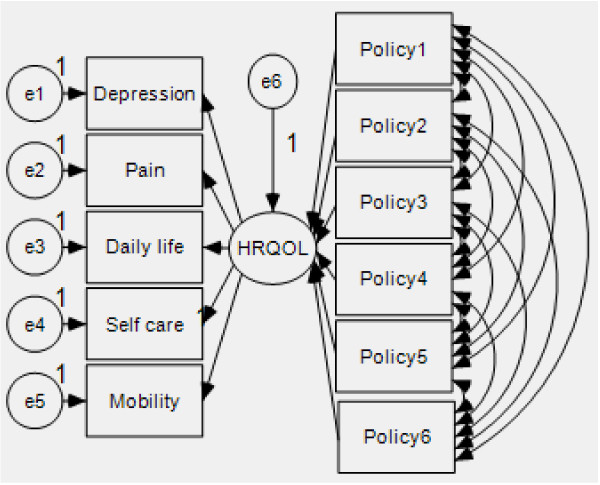
SEM.

Figure 3 shows the SEM, in which HRQOL is used as the latent variable that comprises the five dimensions of EQ-5D. Six policies affect HRQOL, as shown by the arrows. The model fit indices are compared with reference standards, including the ratio, chi-square/degrees of freedom, RMR, RMSEA, GFI, AGFI, NFI, IFI, TLI (NNFI), CFI, PGFI, PNFI, and PCFI. A chi-square value of 37.8, a degree of freedom of 29, and a chi-square/degree of freedom of 1.303 <2 indicate an acceptable model fit. Aside from PGFI that is slightly smaller than the standard value, the other fit indices have met the required values, which imply that the convergent validity of the measurement model can be used for reference.

*First,* Table 4 shows the results of the analysis. The parameter estimates of the five dimensions of EQ-5D and of HRQOL (>1) are positively correlated. Therefore, these five dimensions can reflect the HRQOL of the landless peasants. The C.R. coefficients are all greater than 2 and all probabilities have passed the significance test.

**Table 4:** Analysis results

	**Estimate**	**S.E.**	**C.R.**	**P**
HRQOL ← Policy1	−0.084	0.012	−7.198	***
HRQOL ← Policy6	−0.08	0.011	−7.310	***
HRQOL ← Policy3	−0.065	0.011	−5.258	***
HRQOL ← Policy4	−0.061	0.011	−5.612	***
HRQOL ← Policy5	−0.077	0.012	−6.376	***
HRQOL ← Policy2	−0.076	0.011	−7.146	***
Mobility ← HRQOL	1.000			
Self-care ← HRQOL	1.073	0.049	21.770	***
Daily life ← HRQOL	1.025	0.047	21.589	***
Pain ← HRQOL	1.018	0.049	20.853	***
Depression ← HRQOL	1.014	0.049	20.728	***

Note: ***Indicates the path coefficients are significant when their significance is over 99%.

Note: Policy 1: Land acquisition compensation policy.

Policy 2: Medical insurance policy.

Policy 3: Pension insurance policy.

Policy 4: Employment security policy.

Policy 5: Basic livelihood guarantee policy.

Policy 6: Housing compensation policy.

*Second*, the parameter estimate of daily activities and HRQOL is 1.025, which indicates that an increase in the unit of daily activities raises the HRQOL by 1.018 units. The satisfaction of the landless farmers with the social security policies positively affects their HRQOL. For example, an increase in their satisfaction with the housing compensation policy raises their HRQOL by 0.08 units.

*Third*, among the five dimensions of EQ-5D, daily activities produce the greatest effect on HRQOL based on the parameter estimate (1.025). The difficulty in performing daily activities negatively affects the HRQOL of the landless farmers. Those who are completely unable to perform their daily activities may require long-term care, which further reduces their HRQOL.

*Fourth*, by observing the parameter values, land requisition policy shows the strongest impact on the HRQOL. This result is entirely understandable. The largest difference between landless and common farmers is that the former lost their land, which has a vital role in their survival. Therefore, land acquisition has an enormous effect on the change in HRQOL. If landless farmers are dissatisfied with the compensation system for land acquisition, this dissatisfaction will definitely reduce their HRQOL. When exploring strategies for solutions, improving the implementation of the land acquisition policy is essential.

*Fifth*, by observing the parameter values, employment security policy for landless farmers shows a weak effect on the HRQOL. Employment security aims to provide jobs for landless farmers, ensure their incomes, and meet their needs to maintain daily life. However, considering the special circumstances of landless farmers, they may not completely adjust themselves to the loss of their lands and the need to seek a new job. In addition, employment security may affect the HRQOL in the long term and slow process. Thus, the parameter estimates are smaller compared with other policies.

## Discussion and conclusions

When people mention urbanization in China, labels such as “collectivization of land”, “rural–urban dual structure”, and “household barriers” appear. These labels describe our actual situation. The status quo did not upset us, but turning a blind eye and taking the situation for granted were disturbing. China has formed 10 urban agglomerations, including three national ones (Beijing-Tianjin-Hebei, the Yangtze River Delta, and the Pearl River Delta) and seven with a certain scale (Southern Liaoning, Shandong Peninsula, and so on). The rapid urbanization in China has deprived many farmers of their lands and of the benefits of urbanization. These farmers are often in a disadvantaged position in the land acquisition process. The implementation of social security policies is very important for the long-term and sustainable development of these landless farmers. These social security policies cover the land compensation, health insurance, pension insurance, job security, livelihood security, and basic housing compensation for the landless farmers.

This paper sheds light on the satisfaction of landless farmers with these policies. Although the health conditions of the Chinese population have been given much attention in previous research, the EQ-5D scale has been rarely adopted for measuring the HRQOL of such population. The HRQOL of landless farmers has never been measured by a standardized scale, which presents the research gap that this paper aims to fill. This paper studies the HRQOL of landless farmers from a new perspective by adopting the EQ-5D scale by analyzing the effects of the social security policies on the HRQOL of landless farmers. The main findings of this study are presented as follows:

I. *First*, more than 50% of these landless farmers are in poor or non-serious health conditions, and most of these farmers suffer from anxiety or depression.

II. *Second*, multinomial regression analysis results show that landless farmers can improve their satisfaction with the social security policies. These policies can improve the self-care capacity and capability to perform daily activities as well as reduce the pain, anxiety, or depression of these landless farmers. The satisfaction of the landless farmers with the social security policies can also improve their health condition. However, the effect intensity of these policies may vary among different groups of landless farmers.

III. *Third*, the SEM results show that the satisfaction of the landless farmers with these social security policies positively influences their HRQOL. Among the five dimensions of the EQ-5D scale, daily activities produce the greatest influence on the HRQOL of the landless farmers. Among the six policies, the land acquisition compensation and the employment security policies produce the greatest and weakest influences on the HRQOL of landless farmers, respectively.

The land acquisition compensation policy pays the farmers based on how they have used their lands, which in turn is based on the agricultural land compensation standard. The price of an agricultural land escalates after the land is converted into a construction land. This price may be higher than what the farmers have received from their compensation. After the land requisition, the government usually pays the landless farmers with four compensation fees, namely, land compensation fee, resettlement fee, ground attachments, and young crops compensation fee. The first two are paid to the rural collective economic organizations, whereas the latter two are paid to the owner of the land. Therefore, this policy favors the collective economic organizations than the individual interests of the landless farmers.

The PRC Land Management Law emphasizes that the process must be administrative and compulsory. This process is an absolute government action that collective and individual farmers are forced to follow. Given that the local government executes and sets the standards for the land acquisition, the fairness of the land acquisition compensation policy has become an important issue. Therefore, we suggest, the value-added part of the land and the economic development of the region must be considered when issuing compensations. This must be coordinated with market forces and the development of various resettlement modes. Aside from monetary compensation, the local government may consider adjusting or creating a new agricultural land for the farmers to continue with their primary livelihoods.

The medical insurance policy also significantly affects the HRQOL of landless farmers. The New Cooperative Medical Policy (NCMS)is a medical insurance plan that has been introduced in 2003 to reduce the medical expenditures of Chinese rural residents. However, many rural households are still faced with escalating medical expenses [49] even though this policy has improved the quality and utility of medical services [50-52]. The Chinese government must continue exploring solutions for the improvement of medical services, the reduction of medical expenses, and the expansion of NCMS. We suggest that the government establish a health system that meets the needs of landless farmers. Forming an effective network of health services and medical referral mechanisms and improving the efficient use of medical funds are likewise recommended. The government has to establish a health monitoring body and improve supervision and enforcement mechanisms to strengthen the supervision of the quality and behavioral health services for landless farmers.

The pension policy reduces the concerns of landless farmers on their security after losing their lands. The majority Chinese rural areas mainly rely on the traditional family pension model, which poses some social risks especially after the farmers lose their lands and become exposed to problems in employment and health care. Therefore, a sustainable pension insurance policy must be implemented to promote social stability. We suggest the further improvement of the rural pension security system based on the pattern of social pooling and individual accounts to converge with the pension security system of urban enterprise workers. Moreover, improving the payment standard, level of government subsidies, and pension entitlements in the current pension security system is imperative to gradually narrow the urban–rural difference.

The lowest living standard security and employment security policies must be given special attention. Landless farmers are different from urban residents as their land and living standard securities are lower as compared to those that are enjoyed by urban residents. Therefore, the living standards of these two groups must be balanced. We suggest that the minimum standard of landless farmers be raised, and a special fund and standardize management should be established. Landless farmers also experience more difficulty in securing employment as compared to other unemployed workers given that their skills are rendered useless after the urbanization process. Most of the landless farmers are still in the young and productive age in the labor force. We suggest that the government provide these landless farmers with employment training and information, and encourage entrepreneurship to promote their employment security.

This paper has two limitations. First, this paper only focuses on landless farmers who reside in cities with developed economies. The cities in the Yangtze River Delta region are not balanced in terms of economic development. Future studies must examine the HRQOL of landless farmers who are living in other parts of the Yangtze River Delta region. Second, this paper only focuses on the influence of social security policies on the HRQOL of landless farmers. Future studies must examine the other influencing factors of HRQOL to help adjust the national policies and to improve the efficiency of the government in protecting the welfare and interests of landless farmers. Consent Written informed consent was obtained from the patient for the publication of this report and any accompanying images.

## Competing interests

The authors declare that they have no competing interests.

## Authors’ contributions

YL wrote and revised the manuscript, was responsible for the design of the study, and performed the statistical analysis. WL and WW participated in the design of the study and in the statistical analysis. All authors read and approved the final manuscript.
